# Structural plasticity of green fluorescent protein to amino acid deletions and fluorescence rescue by folding-enhancing mutations

**DOI:** 10.1186/s12858-015-0046-5

**Published:** 2015-07-25

**Authors:** Shu-su Liu, Xuan Wei, Xue Dong, Liang Xu, Jia Liu, Biao Jiang

**Affiliations:** Shanghai Institute for Advanced Immunochemical Studies, ShanghaiTech University, Shanghai, China; Department of Chemistry and Biochemistry, University of Maryland, College Park, USA

**Keywords:** Green fluorescent protein (GFP), Transposon mutagenesis, Amino acid deletions, Protein folding, Chromophore maturation

## Abstract

**Background:**

Green fluorescent protein (GFP) and its derivative fluorescent proteins (FPs) are among the most commonly used reporter systems for studying gene expression and protein interaction in biomedical research. Most commercially available FPs have been optimized for their oligomerization state to prevent potential structural constraints that may interfere with the native function of fused proteins. Other approach to reducing structural constraints may include minimizing the structure of GFPs. Previous studies in an enhanced GFP variant (EGFP) identified a series of deletions that can retain GFP fluorescence. In this study, we interrogated the structural plasticity of a UV-optimized GFP variant (GFP_UV_) to amino acid deletions, characterized the effects of deletions and explored the feasibility of rescuing the fluorescence of deletion mutants using folding-enhancing mutations.

**Methods:**

Transposon mutagenesis was used to screen amino acid deletions in GFP that led to fluorescent and nonfluorescent phenotypes. The fluorescent GFP mutants were characterized for their whole-cell fluorescence and fraction soluble. Fluorescent GFP mutants with internal deletions were purified and characterized for their spectral and folding properties. Folding-ehancing mutations were introduced to deletion mutants to rescue their compromised fluorescence.

**Results:**

We identified twelve amino acid deletions that can retain the fluorescence of GFP_UV_. Seven of these deletions are either at the N- or C- terminus, while the other five are located at internal helices or strands. Further analysis suggested that the five internal deletions diminished the efficiency of protein folding and chromophore maturation. Protein expression under hypothermic condition or incorporation of folding-enhancing mutations could rescue the compromised fluorescence of deletion mutants. In addition, we generated dual deletion mutants that can retain GFP fluorescence.

**Conclusion:**

Our results suggested that a “size-minimized” GFP may be developed by iterative incorporation of amino acid deletions, followed by fluorescence rescue with folding-enhancing mutations.

**Electronic supplementary material:**

The online version of this article (doi:10.1186/s12858-015-0046-5) contains supplementary material, which is available to authorized users.

## Background

The discovery and application of green fluorescent proteins (GFP) have revolutionized biomedical research during the past several decades. GFP was identified in jellyfish *Aequorea victoria* in the 1960s [1] and then isolated and characterized by Prasher *et al.* in 1992 [2]. Soon after this, GFP was adapted as a fluorescent tag to unravel the details of cellular events, such as protein localization, trafficking and interaction [3]. Extensive engineering studies resulted in a panel of fluorescent protein (FP) variants with a wide range of spectral properties [4]. Other properties such as brightness, cytotoxicity and photostability have been also optimized to facilitate the application of FPs.

Notably, considerable efforts have been made to optimize the oligomerization state of FPs. Many of the naturally occurring FPs are dimeric or tetrameric [4]. Oligomerization does not limit the application of GFPs as reporters for gene expression, but may interfere with the native function of fused proteins. Tsien’s group first demonstrated that tetrameric *Discosoma sp.* red fluorescent protein (DsRed) could be engineered to be monomeric [5]. In this study, they first disabled the formation of tetrameric structure by site-directed mutagenesis and then used random mutagenesis to rescue the fluorescence. It is now known that many oligomeric FPs can be converted into monomers by point mutations without appreciable deleterious effects [6].

Oligomerization rarely caused troubles for the native function of GFPs, however it may introduce structural constraints and unfavorably affect the role of GFPs as a genetic tag for biological applications such as protein-protein interaction and subcellular localization. Despite the extensive optimization, the artifacts associated with GFP oligomerization still require careful assessment in each experiment [7]. In addition to traditional strategy, an alternative approach to reducing the structural constraints associated with GFP tag is to develop a size-minimized construct. This is extremely challenging in the case of GFP because it is generally considered as a “compact” protein due to the well-shaped β-can structure [8–10].

The wild-type *A. victoria* GFP is a 27 kDa protein containing 238 amino acids. GFP and all its known variants adopt a β-can structure assembled by 11 antiparallel β-strands [3, 11]. Most strands are connected by small loops consisting of one to four amino acids. Two larger loops appear at positions 129 ~ 143 and 189 ~ 197. The chromophore of GFP is located in the central α helix, surrounded by β-strands. The top and bottom “lids” composed mainly of residues 74–91 and 128–145, respectively [11, 12].

Several early studies have been performed to understand the tolerance of GFP to amino acid deletions. GFP is well tolerant to deletions at N or C terminus [8, 9]. One study showed that the minimal domain required for the fluorescence of GFP contains residues from 2 to 232 [8]. An enhanced version of GFP (EGFP, mutation F64L/S65T) can still be fluorescent with 5 amino acids deletion from N terminus or 10 amino acid deletion from C terminus [9]. However, existing evidence suggested that GFP is very sensitive to deletions at internal positions. Deletions of the two large loops or the two small helices eliminated the fluorescence of EGFP [9]. Targeted deletion analysis of the longest loop (129 ~ 143) in SuperGlo GFP (sgGFP, mutation F64L/S65C/I167T) showed that I128Δ and D129Δ are the only two single deletions that can retain GFP fluorescence [10].

Nevertheless, all early studies focused on analyzing deletions at the termini or large loops of GFP. In recent studies, Jones *et al.* used a transposon-based mutagenesis method [13] to investigate the global structural plasticity of EGFP to amino acid deletions [14]. A series of deletion mutants that can retain GFP fluorescence have been identified and some mutants even exhibited improved cellular fluorescence [14, 15]. These studies shed the light on the important roles of amino acid deletions on GFP fluorescence and indicated that incorporation of amino acid deletions might be a feasible approach to the development of size-minimized GFP construct. In this study, we created a set of GFP_UV_ deletion mutants using a similar approach, characterized their properties in a comprehensive manner and explored the feasibility of rescuing the fluorescence of deletion mutants by introducing folding-enhancing mutations.

## Results

### Transposon-mediated deletion mutagenesis and colony screening

**Fig. 1:**
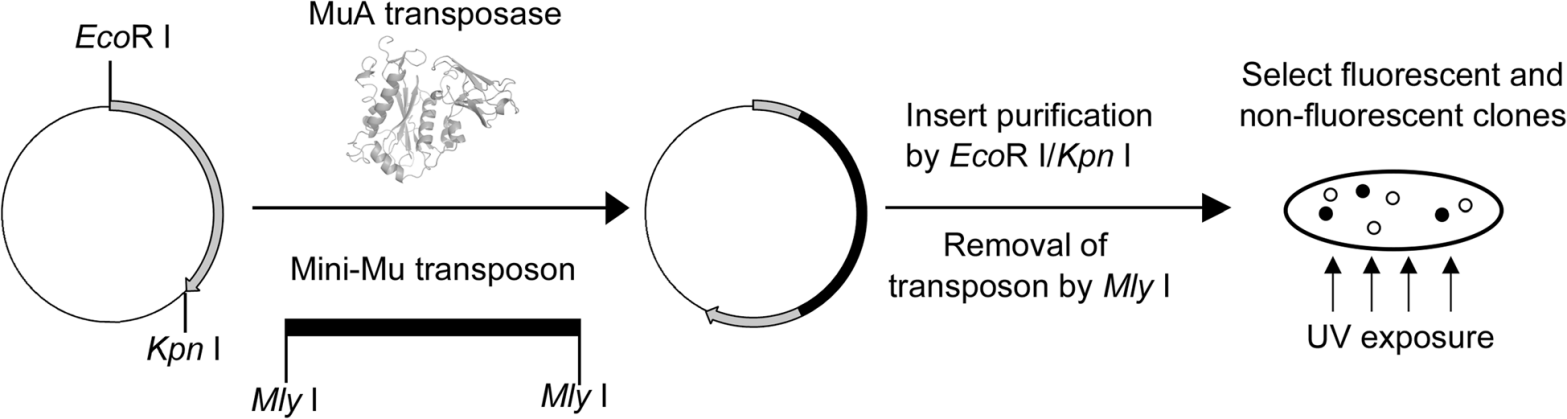
Flow chart for library construction and colony screening

The transposon mutagenesis used in this study was described by Jones *et al.* [13]. It relies on an *in vitro* transposition reaction using MuA transposase and a mini-Mu transposon DNA [16] with engineered *Mly* I recognition site at each end [13]. In the presence of the donor transposon DNA, MuA transposase cleaves the acceptor DNA at a random position in a five nucleotide staggered manner. Following transposon insertion and transformation of the transposition product into bacteria, the five nucleotide overhang is repaired by bacterial DNA repair machinery, leading to duplication of the five nucleotides at the transposon insertion site. Subsequent removal of the transposon DNA using *Mly* I enzyme cleaves four nucleotide from the acceptor DNA at each end of transposon, resulting in a net 3 bp deletion from the target DNA (Fig. 1).

In this study, we performed transposon reaction using *Mly* I Mu transposon [13] as the donor DNA and pGFP_UV_ plasmid as the acceptor DNA. We collected 38,000 individual colonies from the transformation of transposition product, which were sufficient to cover more than 95 % of all possible insertion sites even considering the minor substrate preference of the transposon system [16]. We next used *EcoR* I/*Kpn* I to isolate the transposon inserts within the GFP_UV_ gene (Additional file 1: Figure S1). The final deletion library was obtained by releasing the transposon DNA from pGFP_UV_ plasmid using *Mly* I restriction digestion.

pGFP_UV_ plasmid encodes a GFP variant with enhanced fluorescence under UV light (GFP_UV_) [17]. pGFP_UV_ plasmid constitutively expresses GFP_UV_ protein and thus can be used directly for functional screen. In pilot experiments, we observed that some mutants exhibited considerable difference in fluorescence when grown at different temperature. We chose to perform the fluorescence screening at low temperature (20 °C). Forty fluorescent and 24 non-fluorescent colonies were identified and selected for sequencing for further analysis.

### Sequence analysis of deletion mutants

**Fig. 2:**
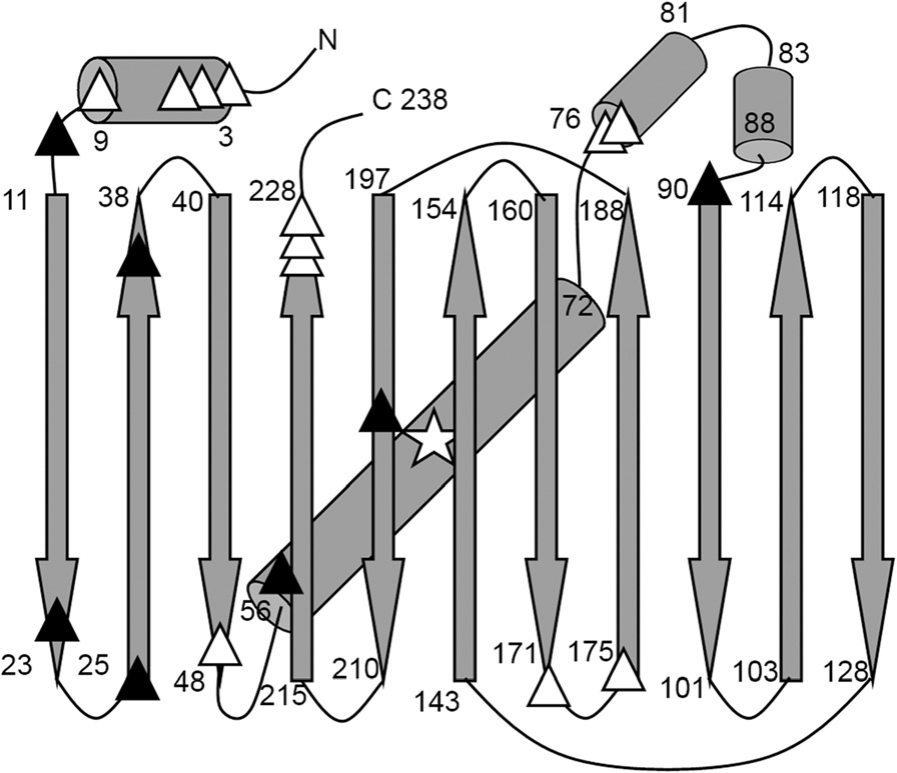
Sequence analysis of identified deletions in GFP_UV_. α-helices and β-strands are shown as cylinders and arrows, respectively. The chromophore is indicated by a star. Deletions leading to fluorescent and non-fluorescent phenotypes are shown as white and black triangles, respectively. Fluorescent mutants include S2/K3➔R, G4Δ, E5Δ, F8/T9➔S, C48Δ, P75/D76➔H, P75Δ, E172Δ, S175/V176➔F, A226Δ, G228Δ and G228/I229➔V (M1Δ mutant is not included due to the deletion of the start codon). Non-fluorescent mutants include G10Δ, D21Δ, G24Δ, E34Δ, P58Δ, P89/E90➔Q and T203Δ

Sequencing results revealed that a small fraction of mutants contained four nucleotide deletion, which presumably resulted from excessive *Mly* I digestion (Additional file 2: Table S1). This result was consistent with previous study [18] and suggested that *Mly* I digestion might require further optimization. In total, we identified thirteen fluorescent and seven non-fluorescent mutants with unique deletions. Of the thirteen fluorescent mutants, eight contained deletions at N- or C-terminus whereas five carried deletions at internal positions. The five internal deletions were all located at the termini of α-helices or β-strands. For the seven deletions that eliminated GFP fluorescence, one was found in the N-terminal region, one was in the middle of β-strand and the rest were all found at the termini of α-helices or β-strands (Fig. 2). It is worth noting that the random nucleotide deletions may occur in two neighboring residues, leading to amino acid mutations in addition to deletions.

### Whole-cell fluorescence and fraction soluble of deletion mutants

**Fig. 3:**
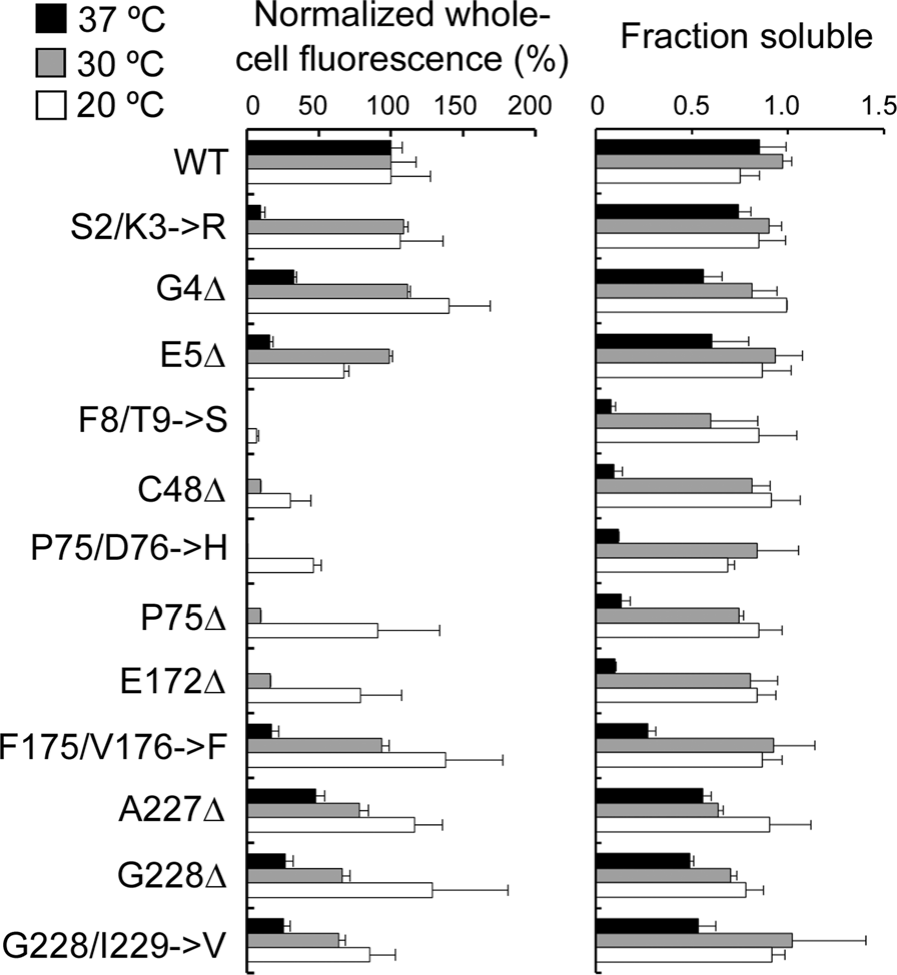
Whole-cell fluorescence and fraction soluble of deletion mutants. The fluorescence of deletion mutants are normalized to that of wtGFP_UV_ under the same temperature. The arbitrary fluorescence of wtGFP_UV_ are 3497, 2620 and 442 under 37, 30 and 23 °C, respectively

In order to quantitatively analyse the identified fluorescent deletion mutants, we characterized their whole-cell fluorescence in the context of pGFP_UV_ plasmid. As temperature may affect the fluorescence of some mutants, whole-cell fluorescence was determined under different temperature: 37, 30 and 20 °C. It was found that, for all temperature, terminal deletions generally retained higher degree of fluorescence compared with internal deletions. The fluorescence of all deletion mutants was compromised at high temperature (37 °C). Decrease of temperature to 30 °C restored the fluorescence of most mutants except F8/T9**➔**S and P75/D76**➔**H. Expression of protein at 20 °C essentially recovered the fluorescence of all mutants (Fig. 3).

The recovery of fluorescence at low temperature suggested that these deletions might impair protein folding, which is a temperature-dependent process. In order to understand the effect of amino acid deletions on protein folding of GFP_UV_, we sought to determine the fraction of soluble GFP_UV_ at different temperature using a previously described method [19]. Wild-type and mutant GFP_UV_ was expressed from pGFP_UV_ plasmid and the bacterial cells were then lysed to assess the GFP present in the entire or soluble fraction of the cell extracts (Additional file 3: Figure S2). The soluble fraction of deletion mutants was clearly temperature dependent (Fig. 3) and plausibly correlated with the whole-cell fluorescence. For example, the mutants with no fluorescence at 37 °C (F8/T9**➔**S, C48Δ, P75/D76**➔**H, P75Δ and E172Δ) had dramatically reduced level of soluble fraction of proteins compared with other mutants. However, factors other than protein folding need be considered to explain the reduced fluorescence of deletion mutants. In one case, P75/D76**➔**H mutant exhibited similar level of soluble fraction of proteins at 30 and 20 °C, but it was only fluorescent at 20 °C. It is known that both protein folding and chromophore maturation of GFP are temperature dependent [20]. Therefore, we intended to purify the deletion mutants and characterize their biochemical and biophysical properties. We chose to analyze the fluorescent mutants with internal deletions. Characterization of these mutants and search of feasible means to rescuing their compromised fluorescence might be the first step toward generating a size-minimized GFP.

### Protein purification and the effect of amino acid deletions on spectral properties

**Fig. 4:**
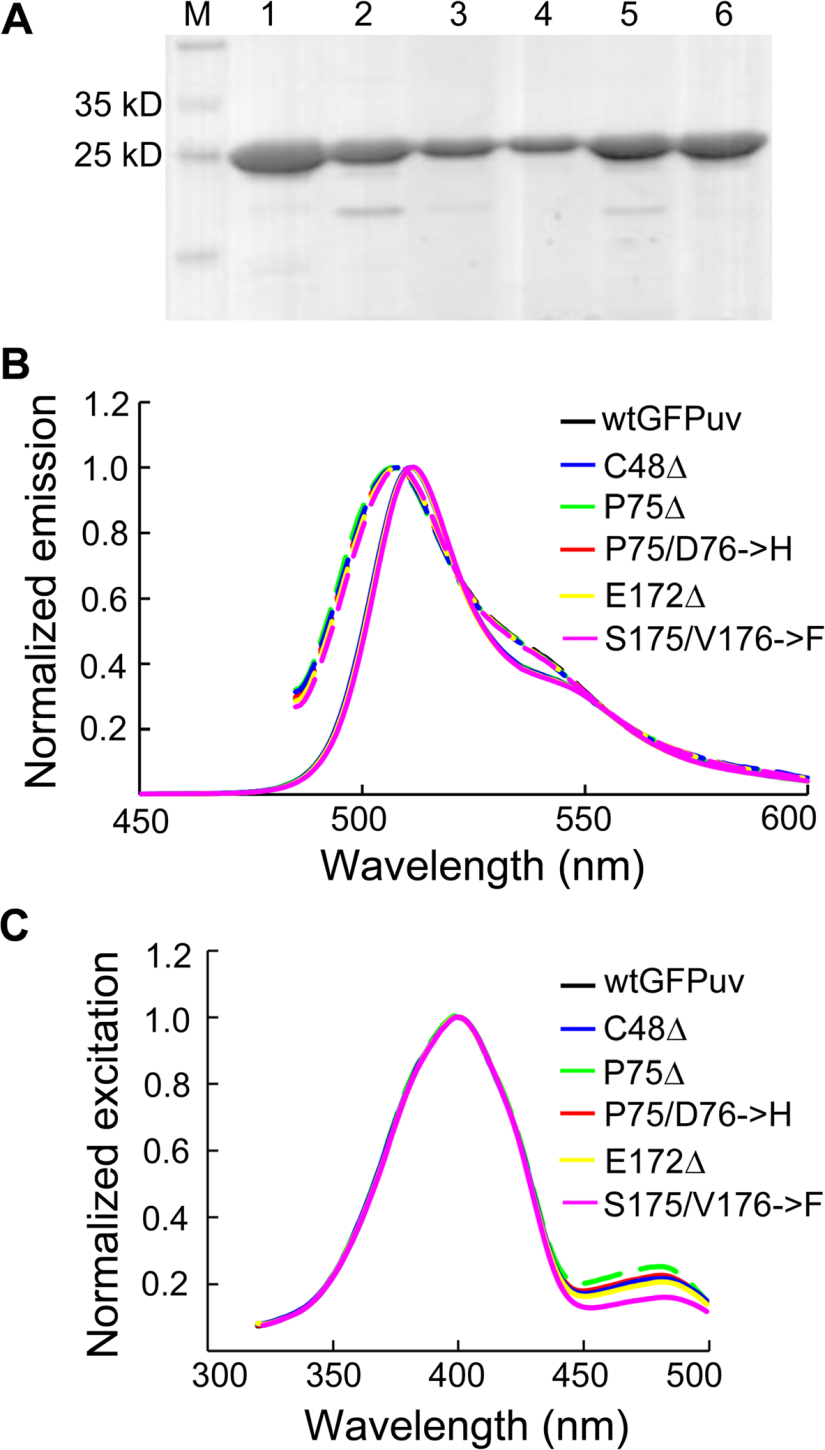
Excitation and emission spectra of purified GPF_UV_ variants. **a** Purified wtGFP_UV_ and deletion mutants, resolved on 12 % SDS-PAGE. M, marker. Lane 1 to 6, wtGFP_UV_, C48Δ, P75Δ, P75/D76➔H, E172Δ and S175/V176➔F, respectively. **b** Normalized excitation spectra. **c** Normalized emission spectra. Note that the spectra of these variants are largely overlapped

Deletion mutants C48Δ, P75/D76**➔**H, P75Δ, E172Δ, S175/V176**➔**F were purified to more than 95 % homogeneity (Fig. 4a). The emission spectra of all variants under 397 and 475 nm excitation can be superimposed with that of wild-type GFP_UV_ (wtGFP_UV_) (Fig. 4b), suggesting that the chromophores of these mutants did not have structural alteration. For excitation spectra, two peaks (397 and 475 nm) were observed for all samples. These deletion mutants showed variations in the intensity ratio between the major and minor peaks. For example, P75Δ showed slightly increased minor peak whereas the minor peak in S175/V176**➔**F mutant was considerably reduced (Fig. 4c). The 395 nm and 475 nm excitation peaks represent neutral and anionic chromophore, respectively [20]. Mutations that alter the ratio of the two chromophore species are frequently found in GFP variants. In one case, enhanced GFP (EGFP) only has a single excitation peak at 475 nm [21] because S65T mutation transform the chromophore to be completely ionized [20]. It is interesting to observe in this study that amino acid deletions, in addition to substitutions, can have impact on the ionization state of chromophore.

We next determined the extinction coefficients at 397 and 475 nm (ε_397_ and ε_475_) and quantum yield of the GFP_UV_ variants. Consistent with the spectra, ε_397_ of all deletion mutant presented no appreciable difference from that of wtGFP_UV_, while modest variation was observed for ε_475_ (Table 1). We also found that all variants have very similar quantum yield to wtGFP_UV_ protein, suggesting that these internal amino acid deletions did not alter the intrinsic brightness of GFP (Table 1). Taken together, these results indicated that the diminished fluorescence of deletion mutants is mainly attributed to the disrupted folding process, rather than intrinsic brightness.

**Table 1:** Extinction coefficient and quantum yield of deletion mutants

	ε_397_	ε_475_	Quantum yield
	(M^−1^ cm^−1^)	(M^−1^ cm^−1^)	(λ_ex_ = 397 nm)
wtGFP_UV_	29600	7500	0.79^[a]^
C48Δ	31600	8500	0.80
P75/D76➔H	29700	6800	0.79
P75Δ	29400	7100	0.79
E172Δ	29300	7600	0.81
S175/V176➔F	30900	5000	0.79

^[a]^Patterson *et al.*, [26]

### Characterization of refolding kinetics and chromophore maturation of deletion mutants

**Fig. 5:**
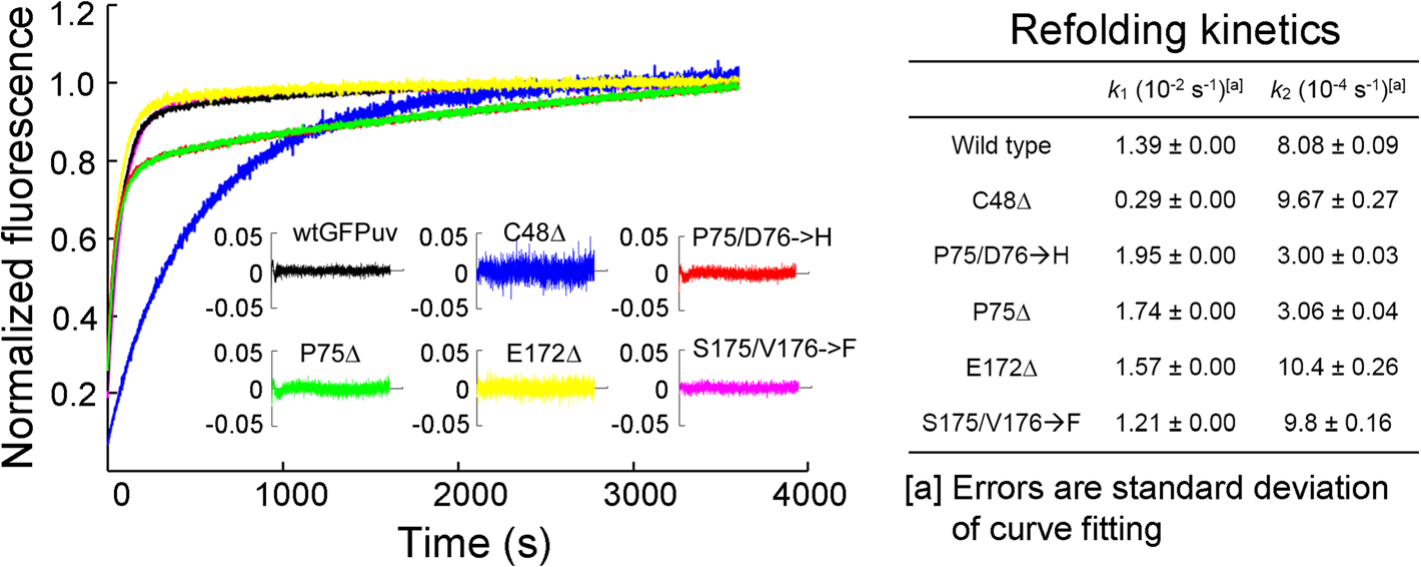
Refolding kinetics. Left panel, kinetic spectra of refolding process. Residuals of curve fitting are shown in the insert. Right panel, fast (*k*_2_) and slow (*k*_2_) folding rates, determined by Prism

As whole-cell lysis study indicated that amino acid deletions may affect protein folding, we next sought to determine the refolding kinetics of deletion variants using purified proteins. Previous study suggested that the refolding process of GFP_UV_ could be fitted equally well with sequential or parallel model [22]. Here we performed refolding experiments using guanidine hydrochloride and recorded the recovered fluorescence as described [22]. The data were fitted into a double exponential equation with a parallel model using Prism 4.0 (Fig. 5). The *k*_1_ and *k*_2_ of wtGFP_UV_ were generally consistent with the reported values [23]. Deletion C48Δ led to notable decrease in *k*_1_ value, suggesting that this deletion was involved in the major folding pathway of GFP. It was also found that P75/D76**➔**H and P75Δ reduced the refolding rate in the slow phase (decreased *k*_2_ value) (Fig. 5). Interestingly, the *cis-trans* isomerization of P75 was proposed as a rate-limiting step in the folding process of GFP [24]. It was also noted that deletion E172Δ impacted neither fast nor slow phase of protein folding. This observation suggested that factors other than protein folding may account for the compromised fluorescence in deletion mutants.

**Fig. 6:**
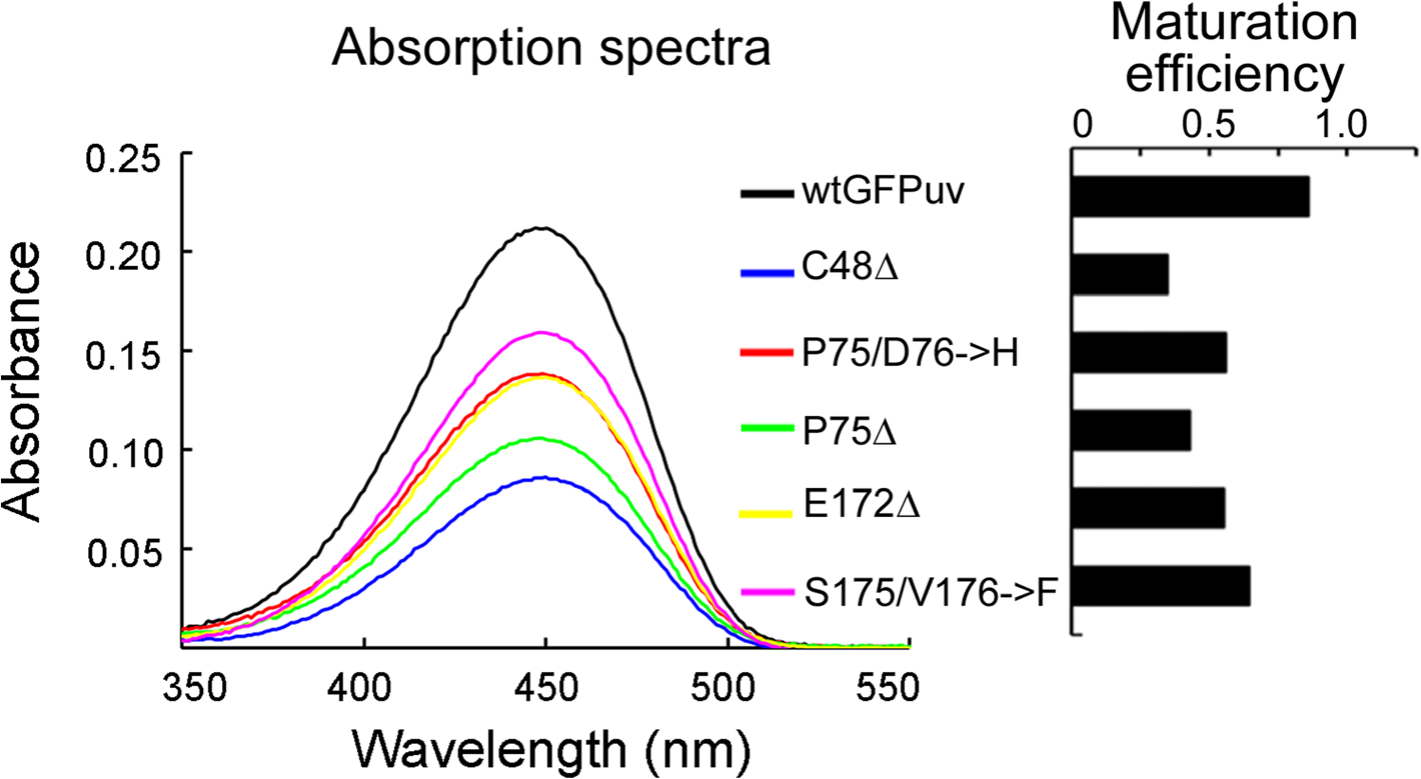
Efficiency of chromophore maturation. Left panel, absorbance of wtGFP_UV_ and variants under base-denatured condition. Right panel, efficiency of chromophore maturation, derived from the absorbance data. Three measurement replicates were performed. Standard deviation is within instrumental error (2 %) and thus not shown in the figure

Instructed by these results, we then analyzed the efficiency of chromophore maturation of deletion mutants. In this experiment, we denatured 7.0 μM protein of each sample and determined their absorbance at 450 nm (Fig. 6). As the extinction coefficient of matured chromophore at 450 nm has been determined in previous study [25], the concentration of matured chromophore can be calculated using Beer’s law. It is evident that all deletions reduced the efficiency of chromophore maturation (Fig. 6). It is worth mentioning that the protein samples used for this experiment was purified at 20 °C. Previous analysis of the whole-cell lysis showed that the five mutant with internal deletions displayed only trace fluorescence but had considerable amount of soluble proteins when expressed at 30 °C. According to the analysis of chromophore, this discrepancy is likely attributed to the impaired chromophore maturation of GFP proteins present in the soluble fraction.

### Fluorescence rescue by folding-enhancing mutations

**Fig. 7:**
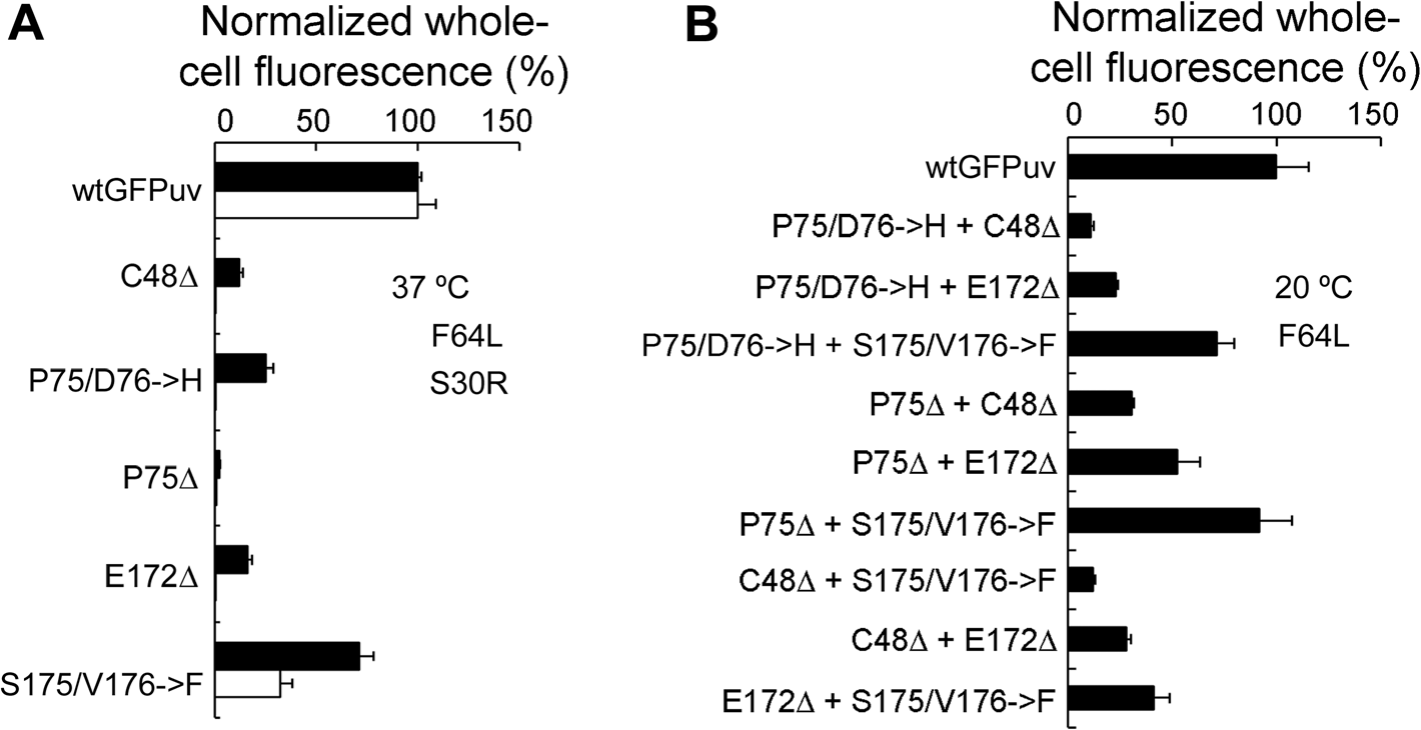
Fluorescence rescue of deletion mutants using folding-enhancing mutations. **a** Rescue of whole-cell fluorescence of single deletion mutants at 37 °C using folding mutations F64L and S30R. The fluorescence of deletion mutants was normalized to that of wtGFP_UV_ carrying corresponding folding mutations. **b** Rescue of whole-cell fluorescence of double deletion mutants at 20 °C by F64L mutation. Three experimental replicates were performed for each sample

Based on the above results, the compromised fluorescence of GFP deletion mutants resulted from disrupted protein folding and chromophore maturation. As a first step toward generating a size-minimized GFP, we speculated whether the defects in these deletion mutants can be restored by folding-enhancing mutations. We chose to test the effect of two previously described folding mutations F64L [26] and S30R [19] on the mutants with internal deletions. Remarkably, F64L recovered a considerable fraction of fluorescence in all variants except P75Δ, whereas S30R only improved the fluorescence of S175/V176**➔**F (Fig. 7a). This result illustrated that folding-enhancing mutations can rescue the compromised fluorescence of deletion mutants and that some mutations may be more effective than others. Encouraged by these results, we attempted to use F64L mutation to rescue mutants carrying multiple internal deletions. We first generated nine dual deletion mutants by combining the identified five internal deletions. As expected, GFP fluorescence was completely abolished by dual deletions, even with prolonged incubation at 4 °C. We next incorporated F64L mutation into these dual mutants. Although F64L could not recover GFP fluorescence at 37 °C, it successfully restored the fluorescence of all mutants to a considerable degree when proteins were expressed at 20 °C (Fig. 7b). To the best of our knowledge, it has not been reported before that GFP mutants carrying multiple internal deletions can retain fluorescence.

## Discussion

Amino acid deletion, insertion and substitution are all important mechanisms for protein evolution in nature. While amino acid substitutions are responsible for the alteration of protein properties in many cases, insertions and deletions (indels) are critical for generating length variation of proteins [27, 28]. Although less commonly used, indels are efficient means for improving protein functions [29, 30]. Established methods for introducing random indels into proteins include RID mutagenesis reported by Murakami *et al.* [31] and RAISE mutagenesis described by Fujii *et al.* [32]. Notably, Jones *et al.* developed a facile transposon-based method for introducing random triplet nucleotide deletions within the full sequence of a given protein [13] and has gone to employ this method to investigate the structural tolerance of TEM-1 β-lactamase to amino acid deletions [33]. This method has been soon adapted for generating nucleotide substitutions with canonical [34] and non-canonical [18, 35–37] amino acids as well as domain insertions [38, 39]. Subsequent modification of this method allowed generation of multiple in-frame codon mutations [40, 41]. In this study, we used this transposon mutagenesis [13] to interrogate the global plasticity of GFP to amino acid deletions.

Early studies suggested that GFP is well tolerated to amino acid deletions at termini [8, 9] but not to those at internal positions [10]. However, recent deletion analysis of EGFP identified a set of internal deletions that can retain or even improve GFP fluorescence [14, 15]. Herein, we generated a deletion library of GFP_UV_ [17] using a similar approach. This GFP variant (mutation F99S/M153T/V163A) has enhanced absorbance under UV light and thus functional screen can be performed directly by visual inspection using UV excitation. We identified 13 fluorescent and 7 non-fluorescent mutants with unique deletions. We first analysed the whole-cell fluorescence of the mutants that retained GFP fluorescence and found that their fluorescence is strongly temperature dependent. Remarkably, several mutants (G4Δ, F175/V176**➔**F, A227Δ and G228Δ) that showed decreased fluorescence at high temperature (37 °C) are even more fluorescent than wtGFP_UV_ when the temperature is reduced to 4 °C. This suggested that these deletions may enhance GFP fluorescence at the cost of protein stability. Further analysis of the fraction of soluble GFP in the whole-cell lysis suggested that the impaired fluorescence of mutants at high temperature was attributed to the disrupted protein folding and that other process need be considered to explain the loss of fluorescence. Characterization of purified GFP proteins carrying internal deletions confirmed the decreased refolding rates in some variants. Additional analysis showed that all internal deletions significantly reduced the efficiency of chromophore maturation. Interestingly, these mutants have the same intrinsic brightness (quantum yield) with wtGFP_UV_. This finding indicated that these mutants used the same autocatalytic process to form GFP chromophore. The present and previous [14, 15] results suggested that it is feasible to recover protein folding and chromophore maturation by introducing beneficial mutations.

Next we attempted to recover the fluorescence of deletion mutants by employing two folding mutations previously identified in GFP. It was shown that F64L mutation successfully rescued the fluorescence of most mutants whereas S30R was less effective. The different roles of F64L and S30R can be explained by their distinct functions in GFP. F64L is a central mutation in close proximity to chromophore and many studies have demonstrated its critical role in protein folding and chromophore maturation [21, 24, 26]. S30R, in contrast, is a distal mutation that enhanced protein folding in an indirect manner [19]. In addition, we analysed the effect of F64L on the non-fluorescent deletion mutants (G10Δ, D21Δ, G24Δ, E34Δ, P58Δ, P89/E90**➔**Q and T203Δ) and found that only G10Δ can recover trace fluorescence at 20 °C (data not shown). It is also worth noting that GFP variants containing F64L mutation can be also used as the starting template for engineering experiments. Previous studies suggested that in the presence of F64L, more deletions may be found to retain GFP fluorescence [14, 15].

Toward the goal of generating a size-minimized GFP, we next set to generate dual deletion mutants and then determined the effects of F64L on these variants. It was found that F64L was capable of rescuing their fluorescence at 20 °C but not that at 37 °C. We also generated GFP_UV_ variants containing triple internal deletions and found that their fluorescence could not be restored by F64L. This result indicated that additional folding mutations or global optimization might be required for compensating the deleterious effects of multiple amino acid deletions. Recent study highlighted the importance of epistasis in protein evolution [42], therefore it may be also interesting to combine the beneficial deletions (G4Δ, F175/V176**➔**F, A227Δ and G228Δ) to explore their interactions.

## Conclusion

We have explored the structural plasticity of GFP to amino acid deletion on a whole-protein scale and showed that compromised fluorescence associated with deletions can be recovered by introducing folding-enhancing mutations. Our results suggested that a “size-optimized” GFP might be developed by iterative deletions of amino acids, followed by fluorescence rescue using folding mutations.

## Methods

### Construction of deletion library

pGFP_UV_ vector (Clontech, Mountain View, CA) was used as the transposon target plasmid for *in vitro* transposition reaction. GFP_UV_ protein carrying a 24 amino acid N-terminal tag is constitutively expressed from this vector. All of the four *Mly* I sites (249, 2334, 2836 and 3322) in pGFP_UV_ vector were removed by site-directed mutagenesis without changing the amino acid sequence (see Additional file 4: Table S2 for primers). The engineered Mu transposon bearing chloramphenicol-resistant gene and *Mly* I sites was constructed as described [13]. Transposon DNA was released from pUC19 vector by *Bgl* II digestion, gel purified and then resolved on a 1 % agarose gel to determine DNA concentration and purity. Transposition reaction was performed in a 20 μL mixture containing 50 mM Tris-acetate, pH 7.5, 150 mM potassium acetate, 10 mM magnesium acetate, 4 mM spermidine, 570 ng of pGFPuv vector, 140 ng of transposon DNA (~1.3 molar excess) and 1 unit of HyperMu MuA transposase (Epicentre Biotechnologies, Madison, WI). The reaction was kept at 30 °C for 4 hrs and stopped by addition of 0.1 % SDS, followed by heat-inactivation at 70 °C for 10 min. The reaction product was transformed into chemically competent GeneHogs *Escherichia coli* cells and plated on LB agar containing 100 μg/mL ampicillin and 10 μg/mL chloramphenicol for selection of pGFP_UV_ vector with transposon insertion. Approximately 38,000 colonies were collected and maxi-prepped to build the pGFP_UV_-MuDel library.

pGFP_UV_-transposon library DNA was digested with *EcoR* I/*Kpn* I, and the 2.0 kb DNA fragment corresponding to transposon-carrying GFP_UV_ gene was re-ligated with the 2.6 kb vector backbone. This purified pGFP_UV_-transposon library was then digested with *Mly* I to remove transposon DNA, leaving a three nucleotide scar at a random position of GFP_UV_. The blunt-end intramolecular ligation was performed in a 20 μL reaction containing 50 mM Tris–HCl, pH7.5, 10 mM MgCl_2_, 10 mM dithiothreitol (DTT) and 0.5 mM ATP, 300 ng DNA, 400 cohesive end units of T4 DNA ligase (NEB). The ligation product was transformed into chemically competent GeneHogs *E. coli* and the transformants were plated on LB agar containing 100 μg/mL ampicillin. In total, 10,000 colonies were collected, and maxi-prepped to build the triplet nucleotide deletion library.

### Screening for deletion mutants

The deletion library DNA was transformed into GeneHogs and the transformants were plated on LB agar supplemented with 100 μg/mL ampicillin at a density of 500 colonies per plate. Transformed cells were grown at 20 °C for 30 h and screened for fluorescence by visual inspection using Spectronics (Westbury, New York) model TC312E UV transilluminator under 310 nm wavelength. Forty fluorescent and 24 non-fluorescent colonies were selected and sequenced for further characterization.

### Liquid culture whole-cell fluorescence

Plasmids encoding wild-type GFP_UV_ (wtGFP_UV_) and deletion mutants were transformed into GeneHogs and plated on LB agar containing 100 μg/mL ampicillin at a density of ~200 colonies per plate. Single colonies with a diameter of ~0.5 mm were inoculated into 2 mL of liquid LB medium supplemented with 100 μg/mL ampicillin and grown at 37, 30 and 23 °C for 14, 18 and 25 h, respectively. Cells were harvested by centrifugation, washed with 500 μL TNG buffer (100 mM Tris–HCl, pH 7.5, 150 mM NaCl and 10 % glycerol) and then resuspended in 100 μL TNG buffer [19]. One milliliter of cell resuspension with an OD_600_ of 0.150 ± 0.003 was prepared for fluorescence assay and the remaining cells in TNG buffer were stored at −20 °C for further experiments. Whole-cell fluorescence was determined using a model F4500 fluorescence spectrophotometer (Hitachi, Tokyo, Japan) as described [19]. The excitation and emission wavelength was set as 397 nm and 509 nm, respectively. Background fluorescence of empty GeneHogs cells was subtracted from each reading. All data were normalized to those of wtGFP_UV_ under the same conditions. Three experimental replicates were performed for each sample.

### Protein expression and fraction soluble

For wild-type and mutant GFP_UV_, 300 μL cell suspension in TNG buffer with an OD_600_ of 0.100 was sonicated by Branson model 450 sonicator (Branson Ultrasonics, Danbury, CT) equipped with a 1/2 inch horn and a 1/8 in. tip. The power output and duty time were both set 50 %. Cell suspension was forced to two sequences of 10 pulse sonication with an interval of 3 min. Following sonication, 150 μL cell lysis was centrifuged at 12,000 *g* for 10 min and the supernatant was transferred into a new tube. SDS loading samples were prepared in a 30 μL solution containing 15 μL crude cell lysis (whole protein) or supernatant (soluble fraction) and 15 μg bovine serum albumin (BSA) protein (Sigma, St. Louis, MO) as an internal standard. Protein samples were resolved in 12 % acrylamide SDS-PAGE gels and analyzed by Image J (http://rsbweb.nih.gov/ij/). GFP expression was quantified based on the density ratio of GFP and BSA bands as described [19]. The fraction soluble of each sample was extrapolated from the density ratio of soluble GFP present in supernatant and overall GFP present in crude cell lysis. Three experimental replicates were performed for each sample.

### Protein expression and purification and determination

wtGFP_UV_ and mutants with internal deletions were sub-cloned into pET28b(+) vector (EMD Chemicals Inc., San Diego, CA) using primers CTAgctagcATGAGTAAAGGAGAAGAACTT (*Nhe* I site in lowercase) and CCCaagcttTTATTTGTAGAGCTCATC (*Hind* III site in lowercase). pET28b vector encoding wild-type and mutant GFP_UV_ with N-terminal His_6_ tags were transformed into *E. coli* BL21 (DE3) cells (Stratagene Inc., La Jolla, CA) and spread on LB agar plates supplemented with 50 μg/mL kanamycin. A single colony was inoculated into 500 mL LB media supplemented with 50 μg/mL kanamycin and grown at 37 °C to an OD_600_ of 0.8. Protein expression was induced with 1 mM isopropyl-β-D-1-galactopyronaside (IPTG) for 12 h at 20 °C. The next day, cells were centrifuged and re-suspended in 25 mL binding buffer (100 mM HEPES, pH 7.5, 5 mM imidazole, 1 mM phenylmethanesulfonyl fluoride, PMSF). Cells were lysed by sonication and then centrifuged at 12,000 g for 30 min. Supernatant was transferred into a new tube and then loaded on to a column pre-packed with 1 mL HisLink resins (Promega Corporation, Madison, WI). The protein-bound resins were washed with 20 mL wash buffer (100 mM HEPES, pH 7.5, 20 mM imidazole) and then eluted with 5 mL elution buffer (100 mM HEPES, pH 7.5, 300 mM imidazole). Purified proteins were concentrated and buffer-exchanged into TNG buffer (100 mM Tris–HCl, pH 7.5, 150 mM NaCl and 10 % glycerol). The concentration of purified proteins were determined using Pierce BCA Protein Assay kit (Thermo Fisher Scientific, Rockford, IL).

### Biophysical properties of deletion mutants

The excitation and emission scan were performed using 6 μg/mL purified proteins in TNG buffer. An emission wavelength of 509 nm was used for excitation scan, while emission scan was performed using excitation wavelengths of 397 nm and 495 nm.

The concentration of matured GFP was determined using “base-denatured” method [25]. Briefly, 7 μM purified proteins were denatured in 0.1 M NaOH for 5 min at 25 °C. Absorption spectra from 300 nm to 600 nm were recorded. The concentration of matured GFPs was calculated from experimentally determined A_447_ and previously reported ε_447_ (44,000 M^−1^ cm^−1^ for denatured GFPs) [25] using Beer’s Law. The efficiency of chromophore maturation was calculated based on the concentrations of matured and overall GFP.

The extinction coefficients of each sample at 397 nm (ε_397_) and 495 nm (ε_495_) were determined using Beer’s Law: *A* = *ε* × *l* × *c*, where *A* is experimentally determined absorbance, *l* is path length and *c* is the concentration of matured GFPs (= total GFP concentration × efficiency of chromophore maturation).

For the measurement of quantum yield, all protein samples were diluted to an OD_397_ of 0.100 and then further diluted 100-fold with water. The emission spectra from 450 nm to 600 nm were scanned using an excitation wavelength of 397 nm. The quantum yield of wtGPF_UV_ has been defined in a previous study as 0.79 [7]. The quantum yield of mutants was calculated by comparing their integrated area of emission spectra with that of wtGPF_UV_. Three experimental replicates were performed for each measurement. The instrumental error was estimated to be 2 %.

### Kinetic refolding experiments

To characterize the refolding ability of the deletion mutants, protein samples were denatured in the following solutions: 20 mM Tris–HCl, pH 7.5, 100 mM NaCl, 1 mM ethylenediaminetetraacetic acid (EDTA), 1 mM DTT, 0.20 mg/mL protein and 6 M guanidine hydrochloride (GdnHCl). Protein unfolding was processed at 25 °C for 24 h. Refolding process was initiated by diluting the denaturation solution 20 fold using the same buffer without GdnHCl. The fluorescence recovery was monitored at 25 °C for 60 min. The refolding data were fitted into a double exponential equation with a parallel refolding model using Prism 4.0 (GraphPad Software Inc., La Jolla, CA).

### Fluorescence rescue by folding-enhancing mutations

Mutations F64L [21] and S30R [19] were introduced into wtGFP_UV_ and deletion mutants by site-directed mutagenesis (see Additional file 4: Table S2 for primers). The whole-cell fluorescence of F64L- or S30R-rescued mutants was assayed as described above. Mutants with double internal deletions were constructed using site-directed mutagenesis and mutation F64L was then introduced into the double deletion mutants. The rescued whole-cell fluorescence was assayed at 20 °C as described above.

## Additional files

Additional file 1: Figure S1. Isolation of transposon inserts inside GFP_UV_ gene. (PDF 179 kb) Additional file 2: Table S1. Sequencing results of 64 selected clones. (PDF 120 kb) Additional file 3: Figure S2. Quantification of the soluble fraction of GFP_UV_ samples using Image J. (PDF 152 kb) Additional file 4: Table S2. Primers used in this study. (PDF 121 kb)

## Abbreviations

GFP: Green fluorescent protein
EGFP: Enhanced green fluorescent protein
GFP_UV_: UV-optimized GFP variant
wtGFP_UV_: Wide-type GFP_UV_
sgGFP: SuperGlo GFP
BSA: Bovine serum albumin
IPTG: Isopropyl-β-D-1-galactopyronaside
PMSF: Phenylmethanesulfonyl fluoride
HEPES: 2-[4-(2-Hydroxyethyl)-1-piperazinyl] ethanesulfonic acid
EDTA: Ethylenediaminetetraacetic acid
GdnHCl: Guanidine hydrochloride
LB: Lysogeny broth
