# Supplementary material for: Structural plasticity of green fluorescent protein to amino acid deletions and fluorescence rescue by folding-enhancing mutations
Source: BMC Biochem. 2015 Jul 25;16:17. doi: 10.1186/s12858-015-0046-5 (PMC4513630; doi:10.1186/s12858-015-0046-5)
Supplement: Additional file 1: Figure S1. — Isolation of transposon inserts inside GFPUV gene. (PDF 179 kb) [file 12858_2015_46_MOESM1_ESM.pdf]

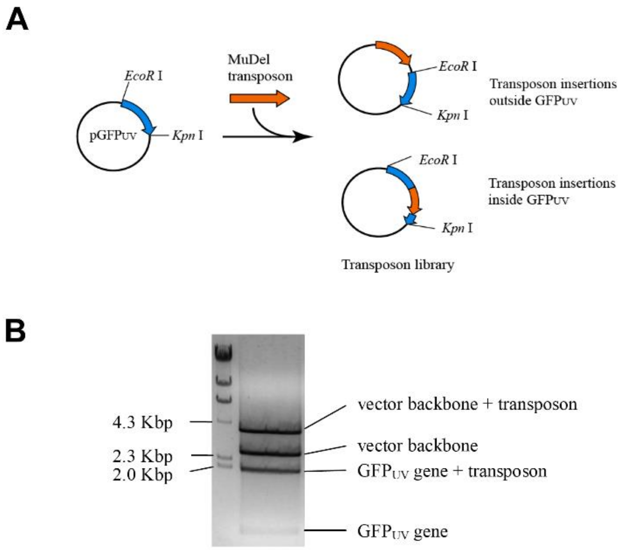

Figure S1. Isolation of transposon inserts inside GFP_{UV} gene. **A**) Possible outcome of transposon insertion. **B**) A 1% agarose gel showing the product from *EcoR* I/*Kpn* I digestion of $pGFP_{UV}$-transposon library. Transposon inserts inside GFP_{UV} gene can be recovered by re-ligating the DNA bands corresponding to "vector backbone" and "GFP_{UV} gene + transposon".
