# Supplementary material for: Structural plasticity of green fluorescent protein to amino acid deletions and fluorescence rescue by folding-enhancing mutations
Source: BMC Biochem. 2015 Jul 25;16:17. doi: 10.1186/s12858-015-0046-5 (PMC4513630; doi:10.1186/s12858-015-0046-5)
Supplement: Additional file 2: Table S1. — Sequencing results of 64 selected clones. (PDF 120 kb) [file 12858_2015_46_MOESM2_ESM.pdf]

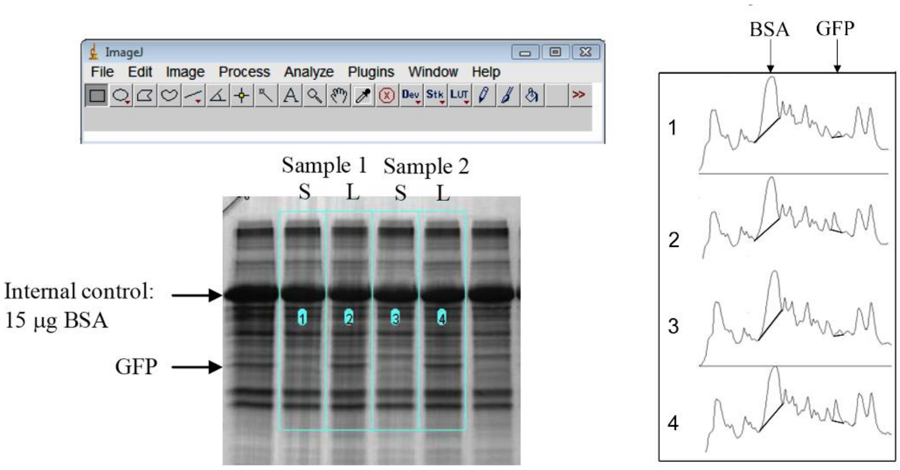

Figure S2. Quantification of the soluble fraction of GFP_{UV} in whole cell lysis using Image J. Bovine serum albumin (BSA, 15 μg) is used as an internal control. S: supernant; L: crude cell lysis.
