# Supplementary material for: Structural plasticity of green fluorescent protein to amino acid deletions and fluorescence rescue by folding-enhancing mutations
Source: BMC Biochem. 2015 Jul 25;16:17. doi: 10.1186/s12858-015-0046-5 (PMC4513630; doi:10.1186/s12858-015-0046-5)
Supplement: Additional file 3: Figure S2. — Quantification of the soluble fraction of GFPUV samples using Image J. (PDF 152 kb) [file 12858_2015_46_MOESM3_ESM.pdf]

Table S1. Sequencing results of 64 selected clones

	[a]Amino acid deletion/change	[b]Nucleotide deletion	Frequency
Fluorescent	M1Δ[c]	1 atg 3	1
	S2/K3→R	1 ATG AGt aaA GGA 12	4
	G4Δ	7 AAA gga GAA GAA 18	11
	E5Δ	10 GGA gaa GAA CTT 21	3
	F8/T9→S	19 CTT Ttc aCT GGA 30	1
	C48Δ	136 TTT ATt tgC ACT 147	1
	P75/D76→H	220 TAT Ccg gAT CAT 231	1
	P75Δ	220 TAT ccg GAT CAT 231	1
	E172Δ	508 AAC ATt gaA GAT 519	1
	S175/V176→F	520 GGA Tcc gTT CAA 531	1
	A227Δ	676 ACT GCT gct GGG 687	2
	G228Δ	676 GCT GCt ggG ATT 687	4
	G228/I229→V	679 GCT Ggg aTT ACA 690	2
Non-fluorescent	G10Δ	22 TTC ACt ggA GTT 33	1
	D21Δ	55 GAT GGT gat GTT 60	2
	G24Δ	64 GTT AAT ggg CAC 75	1
	E34Δ	94 GAG GGt gaA GGT 105	3
	P58Δ	166 CCA TGG cca ACA 177	1
	P89/E90→Q	262 ATG Ccc gAA GGT 273	1
	T203Δ	601 CTG TCG ACA CAA 612	1
Unexpected	No deletions	NA	7
	Stop codon	544 TAtcaA 549	1
	4 bp deletion	NA	13

[a] Triplet nucleotide deletions can occur in one in-frame codon or two neighboring codons, which may result in amino acid mutations.
[b] Deleted nucleotides are lowercased.
[c] This mutant is not subject to further analysis due to deletion in start codon.
