# Supplementary material for: Structural plasticity of green fluorescent protein to amino acid deletions and fluorescence rescue by folding-enhancing mutations
Source: BMC Biochem. 2015 Jul 25;16:17. doi: 10.1186/s12858-015-0046-5 (PMC4513630; doi:10.1186/s12858-015-0046-5)
Supplement: Additional file 4: Table S2. — Primers used in this study. (PDF 121 kb) [file 12858_2015_46_MOESM4_ESM.pdf]

Table S2. Primers used in this study

Name	Sequence (nucleotides to be mutated are lowercased)	Function
Mly1-5'<br>Mly1-3'	TGCATGCCTGCAGGTCGAtTCTAGAGGATCC<br>TCGACCTGCAGGCATGCAAGCTTGGCGTAA	FWD and REV primers for removing *Mly* I site (234)
Mly2-5'<br>Mly2-3'	TTATCTACACGACGGGGAGcCAGGCAACTAT<br>TCCCCGTCGTGTAGATAACTACGATACGGG	FWD and REV primers for removing *Mly* I site (2334)
Mly3-5'<br>Mly3-3'	GTGTCTTACCGGGTTGGAtTCAAGACGATAG<br>TCCAACCCGGTAAGACACGACTTATCGCCA	FWD and REV primers for removing *Mly* I site (2836)
Mly4-5'<br>Mly4-3'	AACGACCGAGCGCAGCGAtTCAGTGAGCGAG<br>TCGCTGCGCTCGGTCGTTCGGCTGCGGCGA	FWD and REV primers for removing *Mly* I site (3322)
F64L-5'<br>F64L-3'	GGCCAACACTTGTCACTACTcttTCTTATGGTGTT<br>AGTAGTGACAAGTGTTGGCCATGGAACAGGTA	FWD and REV primers for introducing F64L mutation
F64L-5'-2<br>F64L-3'-2	ATGGACACTTGTCACTACTcttTCTTATGGTGTT<br>AGTAGTGACAAGTGTCCATGGAACAGGTAGT	FWD and REV primers for introducing F64L into P58Δ
S30R-5'<br>S30R-3'	ATGGGCACAAATTTTCTGTCcgcGGAGAGGGTGAA<br>GACAGAAAATTTGTGCCCATTAACATCACCAT	FWD and REV primers for introducing S30R mutation
C48Δ-5'<br>C48Δ-3'	AAACTTACCCTTAAATTTATCACTACTGGAAA<br>ATAAATTTAAGGGTAAGTTTTCCGTATGTTGC	FWD and REV primers for introducing C48Δ mutation
P75/D76→H-5'<br>P75/D76→H-3'	TCAATGCTTTTCCCGTTATCATCATATGAAAC<br>GATAACGGGAAAAGCATTGAACACCATAAGAG	FWD and REV primers for P75/D76→H mutation
P75Δ-5'<br>P75Δ-3'	TTCAATGCTTTTCCCGTTATGATCATATGAAA<br>ATAACGGGAAAAGCATTGAACACCATAAGAGA	FWD and REV primers for introducing P75Δ mutation
E172Δ-5'<br>E172Δ-3'	TTCAAAATTCGCCACAACATAGATGGATCCGT<br>ATGTTGTGGCGAATTTTGAAGTTAGCTTTGAT	FWD and REV primers for introducing E172Δ mutation
S175/V176→F-5'<br>S175/V176→F-3'	CCACAACATTGAAGATGGATTTCAACTAGCAG<br>ATCCATCTTCAATGTTGTGGCGAATTTTGAAG	FWD and REV primers for S175/V176→F mutation
S175/V176→F-5'-2<br>S175/V176→F-3'-2	TCGCCACAACATAGATGGATTTCAACTAGCAG<br>ATCCATCTATGTTGTGGCGAATTTTGAAGTTA	FWD and REV primers for introducing S175/V176→F mutation into E172Δ mutant
